# Selective Immobilization of Fluorescent Proteins for the Fabrication of Photoactive Materials

**DOI:** 10.3390/molecules24152775

**Published:** 2019-07-30

**Authors:** Ana I. Benítez-Mateos, Ehsan Mehravar, Susana Velasco-Lozano, Radmila Tomovska, Luca Salassa, Fernando López-Gallego

**Affiliations:** 1 Heterogeneous biocatalysis group, CICbiomaGUNE, Edificio Empresarial “C”, Paseo de Miramón, 182, 20014 Donostia-San Sebastián, Spain; 2 POLYMAT and Departamento de Química Aplicada, Facultad de Ciencias Químicas, University of the Basque Country, UPV/EHU, 20018 Donostia-San Sebastián, Spain; 3 Heterogeneous biocatalysis laboratory, Instituto de Síntesis Química y Catálisis Homogénea (ISQCH), CSIC-Universidad de Zaragoza, C/Pedro Cerbuna 12, 50009 Zaragoza, Spain; 4 Ikerbasque, Basque Foundation for Science, Maria Diaz de Haro 3, 48013 Bilbao, Spain; 5 Fakultatea, Euskal Herriko Unibertsitatea, UPV/EHU, 20080 Donostia-San Sebastián, Spain; 6 Donostia International Physics Center (DIPC), Paseo Manuel de Lardizabal 4, 20018 Donostia-San Sebastián, Spain; 7 ARAID, Aragon foundation for Science, 50018 Zaragoza, Spain

**Keywords:** protein immobilization, polypeptide-tags, fluorescent proteins, upconverting, nanomaterials

## Abstract

The immobilization of fluorescent proteins is a key technology enabling to fabricate a new generation of photoactive materials with potential technological applications. Herein we have exploited superfolder green (sGFP) and red (RFP) fluorescent proteins expressed with different polypeptide tags. We fused these fluorescent proteins to His-tags to immobilize them on graphene 3D hydrogels, and Cys-tags to immobilize them on porous microparticles activated with either epoxy or disulfide groups and with Lys-tags to immobilize them on upconverting nanoparticles functionalized with carboxylic groups. Genetically programming sGFP and RFP with Cys-tag and His-tag, respectively, allowed tuning the protein spatial organization either across the porous structure of two microbeads with different functional groups (agarose-based materials activated with metal chelates and epoxy-methacrylate materials) or across the surface of a single microbead functionalized with both metal-chelates and disulfide groups. By using different polypeptide tags, we can control the attachment chemistry but also the localization of the fluorescent proteins across the material surfaces. The resulting photoactive material formed by His-RFP immobilized on graphene hydrogels has been tested as pH indicator to measure pH changes in the alkaline region, although the immobilized fluorescent protein exhibited a narrower dynamic range to measure pH than the soluble fluorescent protein. Likewise, the immobilization of Lys-sGFP on alginate-coated upconverting nanoparticles enabled the infrared excitation of the fluorescent protein to be used as a green light emitter. These novel photoactive biomaterials open new avenues for innovative technological developments towards the fabrication of biosensors and photonic devices.

## 1. Introduction

Fluorescent proteins (FPs) emerged two decades ago as a groundbreaking tool to shine light on biological processes. Since the discovery of the green fluorescent protein from *Aequorea victoria* jellyfish in 1962 by Shimomura [1] that revolutionized cell biology, the scientific community has created an arsenal of fluorescent variants that covers the whole visible spectrum. In this context, one can find FPs with almost any excitation and emission requirements the application demands [2,3,4]. This has been possible due to the engineering of the protein chromophores by mutating some of the amino acids forming them. Matching the excitation/emission ranges of FPs, biomolecular interactions can be better understood by exploiting FRET (Förster Resonance Energy Transfer). Moreover, FPs are very useful to study both qualitative and quantitative dynamics of biomolecules in different environments. Both genetic and chemical fusions of these photoactive proteins with other biomolecules allow the understanding of many biological interactions that are crucial for cellular processes. In this sense, biosensors based on soluble FPs have been extensively exploited as pH indicators [5,6,7], ROS (Reactive Oxygen Species) detectors [8,9,10], and temperature probes [11,12]. Those studies have been crucial for a better understanding of biological disorders.

Beyond the outstanding application of FPs in fundamental biology, they have also been incorporated into different solid materials to fabricate photoactive systems such biosensors and optoelectronic surfaces that open innovative technological avenues. Although the applications of immobilized FPs on solid materials have been much less studied, there are some examples of FPs as pH sensors [13], mechanical damage reporters [14], and toxicity indicators [15,16]. Recently, the immobilization of superfolder yellow fluorescent proteins immobilized on porous agarose microbeads have been used as ratiometric pH sensors for *in operando* biocatalysis studies. Here, the authors monitored the intraparticle pH gradients created by the action of an acylase co-immobilized with the fluorescent protein [17]. On the other hand, immobilized FPs have been used as bio-emitters to fabricate hybrid white-emitting LEDs (Light Emitting Diodes) [18]. In these devices, different FPs emitting both green and red light are combined with UV-LEDs to generate white light. To this end, FPs were entrapped into different organic polymers that coat the surface of commercial UV- or blue-LED chips to enable a bottom-up transfer energy process to generate the target white light. These white bio-LEDs were proven to be more robust than other down converting species such as carbon nanodots, luminescent polymers, and small dyes [19].

Nevertheless, the immobilization of fluorescent proteins is not trivial, since the 3D-structure of these proteins is often distorted during the immobilization process, consequently limiting their performance as photoactive molecules. The vast majority of biomaterials functionalized with FPs involve protein entrapment and physical adsorption rather than irreversible immobilization through covalent bonds. Normally, FPs are randomly immobilized controlling neither the protein residues involved in the attachment nor the protein orientation upon immobilization. Hence, spatial organization of FPs across the material surface is rarely tuned, despite it being demonstrated that chromophore arrangement is crucial to guarantee the efficiency of the photoactive materials [20,21]. The lack of control over immobilization chemistry, orientation and spatial organization of FPs frequently explains the low lighting efficiency and stability of some of the current bio-photoactive materials. These aspects of protein immobilization can be addressed by tuning the surface of the solid materials, the immobilization conditions and the surface of the proteins. The stability of immobilized fluorescent proteins relies on the chemistry through which they are immobilized. Fluorescence anisotropy studies at the single particle level reveal that superfolder green fluorescent proteins are significantly more thermostable when are immobilized through a multivalent attachment based on irreversible short bonds. Our group has previously demonstrated that fluorescent proteins can be immobilized on porous agarose microparticles controlling their spatial organization [22]. Different patterns of spatial organization have been achieved using His-tagged FPs in presence of immobilization competitors. However, such competing molecules sometimes significantly diminish the immobilization yields and consequently the protein loadings, resulting in less bright materials.

To address the challenges posed by the immobilization of FPs, herein we have tuned the immobilization of several fluorescent proteins on a selection of solid materials with the aim of fabricating biomaterials with potential photonic applications. We exploited a toolbox of plasmids, previously developed by our group [23], to produce different variants of FPs tagged with polypeptide tags (His-, Cys- and Lys-tags) to enable their immobilization to a variety of both organic and inorganic materials. This work reports that the genetic reprogramming of the N-terminus of FPs is instrumental to guide both the immobilization and the spatial distribution of these proteins across the surface of materials of different nature and with different physico-chemical properties.

## 2. Results and Discussion

### 2.1. Selective Immobilization of His-Tagged Fluorescent Proteins on Cobalt-Activated Materials

In a previous work, we developed a tool-kit of plasmids that encode superfolder green fluorescent protein (sGFP) tagged with different N-terminal polypeptides: poly-histidine (His-sGFP), poly-cysteine (Cys-sGFP) and poly-lysine (Lys-sGFP) [23]. All these tagged proteins were heterologously overexpressed in *Escherichia coli*. Furthermore, a red fluorescent protein (RFP) tagged with six histidines at its N-terminus (His-RFP) [24] was likewise overexpressed (Figure S1).

The selective immobilization of His-tagged fluorescent proteins on cobalt-activated matrices was demonstrated decades ago [25] and more recently it has been reported how this immobilization chemistry can control the spatial organization of tagged proteins across the porous surface of microspheres [22]. In this work, we have exploited these His-tagged variants to fabricate fluorescent materials based on graphene hydrogels. We have prepared porous composite structures by combining reduced graphene oxide (rGO) nanoplatelets and latex nanoparticles functionalized with epoxy groups (rGOe) by a simple self-assembly procedure. Then, epoxy groups were partially functionalized with cobalt-chelates (rGOe-Co^2+^) to enable the selective and irreversible immobilization of different His-tagged fluorescent proteins. Firstly, the protein was attached to rGOe-Co^2+^ through the His-tag in a site-directed manner, and then the remaining epoxy groups of the material were exploited to irreversibly immobilize the oriented protein [26,27]. His-RFP was quantitatively immobilized on rGOe-Co^2+^, although similar results were found when the graphene material lacked the cobalt-chelates (rGOe) (Table 1). Similarly, >97% of untagged sGFP was also immobilized on both rGOe and rGOe-Co^2+^ (Figure S2). These results suggest some unspecific interactions between the proteins and the graphene-based materials. That unspecific immobilization might be mediated by hydrophobic forces but also driven by random and irreversible interactions between the most reactive and exposed Lys, Ser, His, and Tyr of the FP and the epoxy groups of the composite surface.

To study the effect of the epoxy groups on the irreversibility of the immobilization, we further immobilized His-RFP on rGOe composites with different levels of epoxy activation. The densities of the epoxy groups in rGOe materials were: 0.03 (high); 0.02 (medium); and 0.01 (low) mg of epoxy groups per m^2^ of composite material. In order to obtain quantitative immobilization, we needed the highest activation degree tested. In addition, SDS-PAGE analysis demonstrated that materials activated with medium-high epoxy density fully retained the bound proteins even after boiling the samples for 5 min (Figure S3). On the contrary, the bound His-RFP was partially eluted after boiling when the graphene hydrogel was activated with low epoxy densities. These insights demonstrate that epoxy groups establish covalent and irreversible attachments with the immobilized protein. Furthermore, rGOe-Co^2+^ enabled higher His-RFP loads than rGOe, indicating that cobalt-chelates are driving the immobilization of His-tagged proteins to some extent (Table 1). In summary, the activation of rGO with both epoxy and cobalt-chelate groups at a suitable density enables an efficient, irreversible immobilization of both tagged and non-tagged fluorescent proteins.

**Figure 1:**
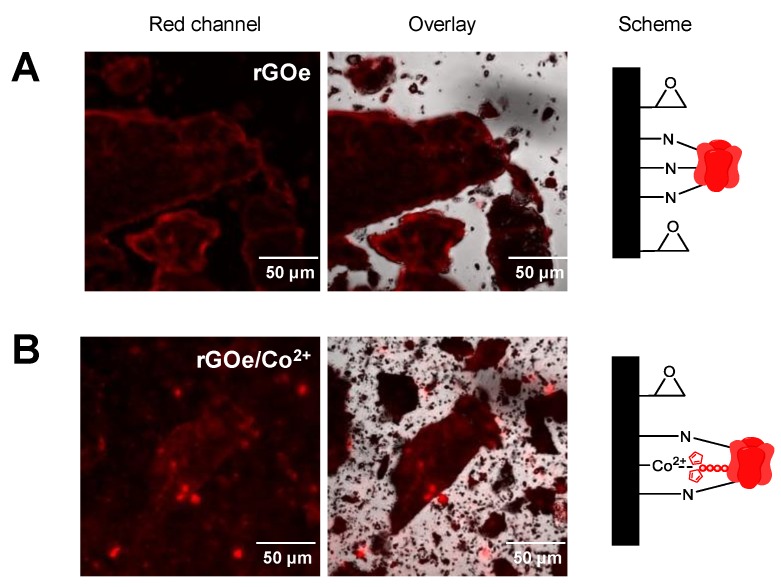
CLSM images of His-RFP immobilized on graphene-based carriers: (**A**) rGOe; (**B**) rGOe-Co^2+^. Both carriers were loaded with 1mg_protein_ × g^−1^_carrier._ From left to right: red channel (λ_ex_: 561 nm, λ_em_: LP565 nm), overlay of fluorescence and brightfield signals, and the scheme of the protein immobilization.

Beyond characterizing the immobilization of fluorescent proteins on this type of graphene-based carrier, we also studied the spatial distribution of His-RFP across the surface of rGO with different functionalizations. In spite of being an opaque material, we could detect the fluorescence underlying the immobilized His-RFP on the surface of rGO flakes by CLSM (Confocal Laser Scanning Microscopy). Random immobilization through unspecific absorption and further irreversible attachment on rGOe localizes the protein at the edges of the particles (Figure 1A), while rGOe-Co^2+^ concentrates the His-tagged protein into bright spots along the flakes (Figure 1B). These different spatial organizations are indications that proteins become primarily immobilized through different mechanisms; unspecific, likely hydrophobic, interactions with rGOe and specific metal coordination bonds between the His-tag of the protein and the cobalt-chelates of the carrier.

**Figure 2:**
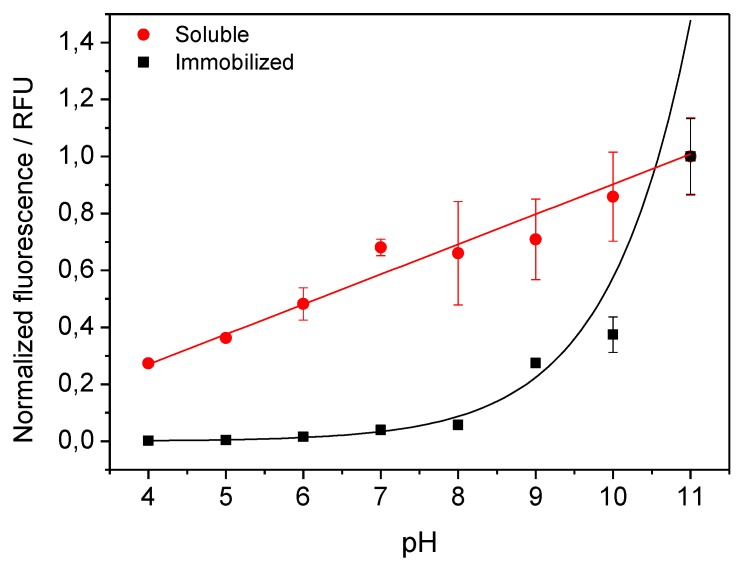
Dynamic range of the fluorescence of soluble and immobilized His-RFP at different pHs. The experimental data of soluble His-RFP were fitted by using a simple linear regression: *y* = a + b*x* (a= −0.15 and b = 0.105). In case of immobilized His-RFP, the experimental data were adjusted to an exponential growth function with constant parameters: *y* = ab*^x^* (where a = 4.6 × 10^−5^ and b = 2.57).

Solid materials functionalized with fluorescent proteins have a variety of technological applications, including as pH-sensors. Herein, we have harnessed the pH-response of RFP to illustrate the capacity of His-RFP immobilized on rGOe-Co^2+^ to sense pH. At acidic pH, the protonation of the chromophore causes contraction of the π conjugation system, resulting in the instability of the chromophore that decreases the fluorescence quantum yield [28]. It has also been reported that some red fluorescent proteins may show a higher stabilization of the cis-protonated chromophore at alkaline pH [6]. Figure 2 shows that the dynamic pH range of immobilized fluorescent protein is narrower than that of the soluble RFP but can still be used as pH sensor for alkaline pH. In fact, the immobilized RFP is significantly more sensitive at alkaline pH, since a change of two pH units, from 11 to 9, resulted in an 80% decrease in fluorescence intensity, while that of the soluble enzyme only decreased 30% over the same pH change. These types of immobilized RFP that respond to alkaline pH might be integrated into different sensing devices. Unlike recently developed pH-sensors based on immobilized yellow fluorescent protein [17], the alkaline pH-dynamic range of His-RFP immobilized on graphene-based materials would be very useful to measure enzymes that turn the reaction media more basic.

### 2.2. Direct and Irreversible Immobilization of Cys-Tagged Fluorescent Proteins on Epoxy-Activated Carriers

**Figure 3:**
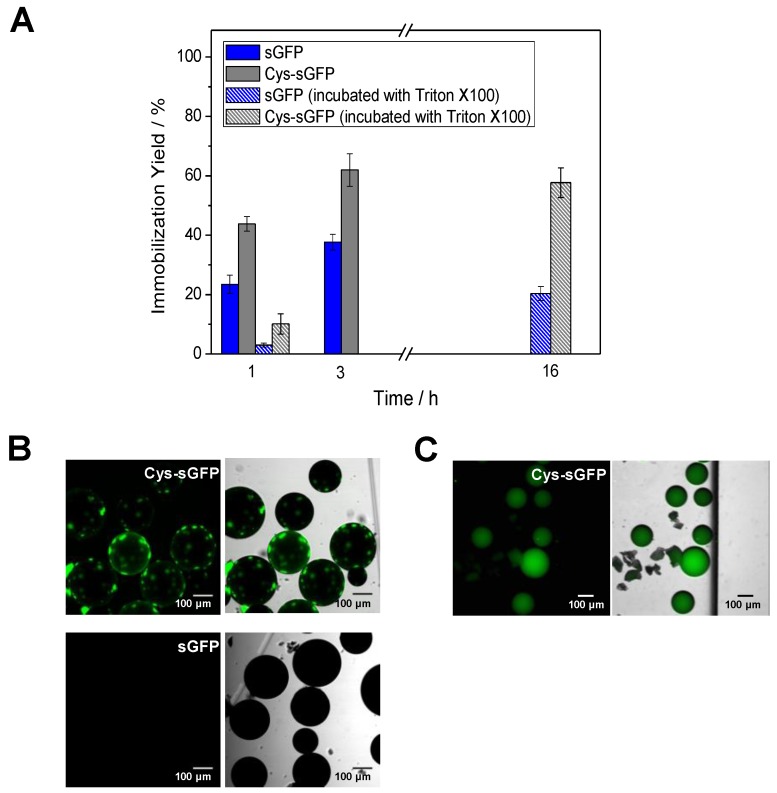
Immobilization of Cys-sGFP on Purolite^®^. (**A**) Immobilization course of sGFP and Cys-sGFP on the epoxy-activated carrier. The yields were calculated by measuring the fluorescence of the supernatants after immobilization (Section 3.5). (**B**) CLSM imaging of the biomaterial after washing steps with Triton X-100. (**C**) CLSM imaging of the biomaterial as a result of incubation with Triton X-100 during the protein immobilization step. Left columns, images of fluorescence (λ_ex_: 488 nm, λ_em_: LP505 nm); right columns, overlay of fluorescence and brightfield signals. The protein immobilization scheme is the same as the one depicted in Figure 1A.

Among the peptide tags comprising the toolbox herein exploited, the genetic fusion of fluorescent proteins to a polycysteine tag enables their one-step immobilization onto commercially available carriers activated with epoxy groups. The commercial resin Purolite^®^ ECR8204, activated with epoxy groups, was utilized to test the binding efficiency of a Cys-tagged green fluorescent protein (Cys-sGFP). The immobilization yield of the tagged protein after 3 h of incubation was 60%, while 40% of its untagged version was also immobilized on the same carrier (Figure 3A). The partial immobilization of the untagged sGFP points out some unspecific interactions between the protein and the carrier surface. In order to decipher the origin of such unspecific interactions, we washed the carriers with detergent (Triton X-100) upon the immobilization to elute those proteins hydrophobically bound to the carriers. After this washing step, the untagged protein was completely eluted from the carrier, since practically no fluorescence signal was detected under the microscope. On the contrary, some patches of Cys-tagged protein still remained bound upon detergent incubation (Figure 3B). This observation demonstrates that the Cys-tag was able to establish a covalent and irreversible interaction with the epoxy groups of the carrier surfaces. After these results, we tried to immobilize the protein on the same carrier in presence of Triton X-100 to accomplish the preparation of the biomaterial in just one step. Despite slowing down protein immobilization, 60% of Cys-sGFP was attached while the untagged version reached only 20% of immobilization yield (Figure 3A). Moreover, the distribution of the Cys-tagged protein across the porous carrier was homogeneous (Figure 3C). This could be explained by a lower immobilization rate when hydrophobic interactions were minimized due to the presence of Triton X-100. Therefore, the introduction of cysteine tags followed by detergent downstream washes or in presence of a detergent allows the direct immobilization of proteins on ready-to-use carriers like methacrylate-based beads activated with epoxy groups that can be supplied by several companies.

### 2.3. Genetically Programmed Spatial Distribution of Tagged Proteins. Co-Immobilization of His- and Cys-Tagged Fluorescent Proteins

**Figure 4:**
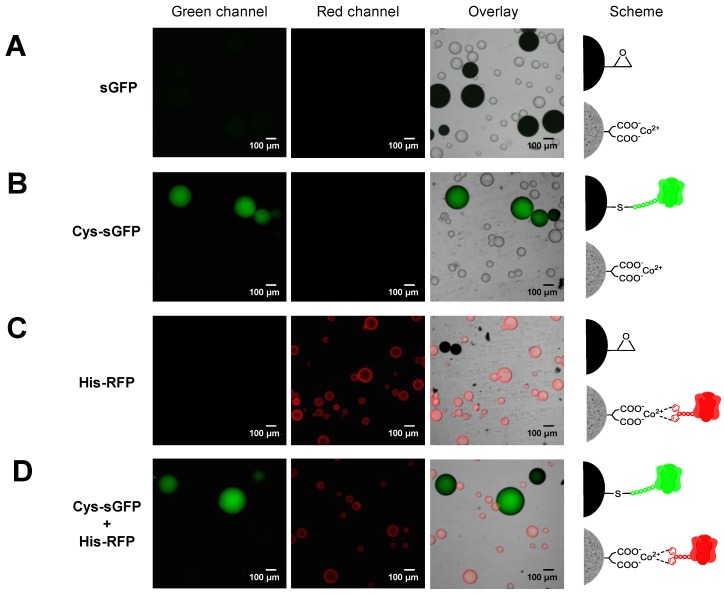
CLSM imaging of the tag-driven selective immobilization of FPs on Purolite^®^ and TALON^®^. (**A**) Untagged sGFP was incubated with both Purolite^®^ and TALON^®^, but no immobilization was detected. (**B**) Cys-sGFP was incubated with both Purolite^®^ and TALON^®^, but only immobilized on Purolite^®^ by irreversible covalent bonds. (**C**) His-RFP was incubated with both Purolite^®^ and TALON^®^, but only immobilized on TALON^®^ by metal coordination bonds. (**D**) Cys-sGFP and His-RFP were incubated with both Purolite^®^ and TALON^®^. Cys-sGFP and His-RFP were selectively immobilized on Purolite^®^ and TALON^®^, respectively. From left to right: green channel (λ_ex_: 488 nm, λ_em_: filter LP505 nm), red channel ((λ_ex_: 561 nm, λ_em_: filter LP565 nm), overlay of fluorescence and brightfield signals, and the scheme of the protein immobilization.

Two or more different proteins can be spatially organized by immobilizing them onto two different carriers or by co-immobilizing them onto the same one. In both cases, one-pot protocols are desired to speed up the preparations of the immobilized proteins. To this aim, the different immobilization chemistries must be orthogonal, selective and compatible to guarantee that each protein is suitably immobilized as designed. Herein, we demonstrated that the immobilization of Cys-tagged proteins on methacrylate-based microbeads activated with epoxy groups (Purolite^®^) is orthogonal with the immobilization of His-tag proteins on commercial agarose-based microbeads activated with cobalt-chelates (TALON^®^). When both fluorescent proteins were immobilized in one-pot, the specific immobilization efficiency was similar to the one obtained when the two proteins were immobilized separately. In one-pot, His-RFP was quantitatively immobilized on TALON^®^, but as expected, less than 50% of the offered Cys-sGFP was immobilized on Purolite^®^ (Figure S4A). As a control, untagged sGFP was incubated with both carriers and no protein immobilization was detected, neither on Purolite^®^ nor TALON^®^ by fluorescence microscopy (Figure 4A). Hence, these tags are orthogonal and allow the one-pot selective immobilization of two different proteins onto two different carriers, achieving a unique spatial organization. In different pots, we observed that the Purolite^®^ carrier specifically binds and uniformly distributes Cys-sGFP across its porous surface (Figure 4B), while TALON^®^ microspheres selectively immobilize His-RFP, mainly localizing the protein at the outer surface of the carrier (Figure 4C). Remarkably, when both proteins were incubated with both carriers in one pot, Cys-sGFP was selectively immobilized on Purolite^®^ and His-RFP was immobilized specifically on TALON^®^ (Figure 4D). Furthermore, SDS-PAGE proved the covalent and irreversible attachment of Cys-sGFP on Purolite^®^ and the reversible binding of His-RFP to the TALON^®^ carrier (Figure S4B). Cys-sGFP was irreversibly immobilized through covalent bonds with the epoxy groups of Purolite^®^. This chemistry relies on the nucleophilic attack of thiols from Cys to the epoxy groups on the carrier surface, yielding irreversible thioether bonds between the protein and the materials. On the contrary, the His-tag of His-RFP establishes reversible cobalt-chelates with the TALON^®^ microbeads that are broken under denaturing conditions.

Instead of compartmentalizing two fluorescent proteins on two different microparticles, the proteins may also be co-localized across the microstructure of the same particle. To that aim, we need to use a heterofunctional carrier that displays different reactive groups able to immobilize the different proteins through different chemistries. Although this concept has been previously exploited by our group [23,29,30,31], in this work we have genetically programmed two fluorescent proteins to be orthogonally co-immobilized on the same surface activated with two different highly selective groups for two different peptide tags. As proof of concept, Cys-sGFP and His-RFP were co-immobilized on agarose beads activated with disulfide and cobalt-chelate groups (AG-Co^2+^/S). To this end, we prepared this heterofunctional carrier according to the protocol already published by our group (see materials and methods) [23]. Immobilization yields were comparable to those found for the isolated immobilization of each protein; 90% for the His-RFP immobilized on AG-Co^2+^/S by metal coordination bonds and 60% for the Cys-sGFP attached to AG-Co^2+^/S by disulfide bonds (Figure S5A). In both cases, the protein-carrier bonds are reversible and can be reverted by incubation with reducing agents [23] and imidazole, respectively. In this work, the samples were analyzed by SDS-PAGE (Figure S5B). This is possible because the thiols from the Cys-tag perform a thio-disulfide exchange with the disulfide groups of the carrier, yielding reversible disulfide bridges between the protein and the support. On the other hand, the His-tag establishes the well-known reversible coordination interactions with the cobalt-chelate groups that drive the immobilization. The untagged proteins were unproductively immobilized on these heterofunctional carriers, while both Cys- and His-tags were successfully and selectively immobilized.

**Figure 5:**
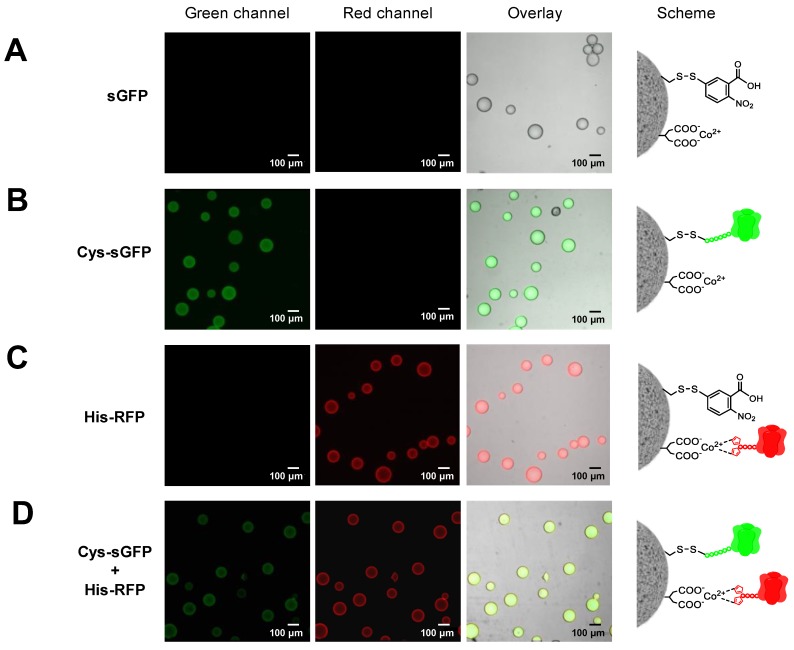
CLSM imaging of the tag-driven selective co-immobilization of FPs on AG-Co^2+^/S. (**A**) Untagged sGFP was incubated with AG-Co^2+^/S, but no immobilization was detected. (**B**) Cys-sGFP immobilized on AG-Co^2+^/S by reversible disulfide bonds. (**C**) His-RFP immobilized on AG-Co^2+^/S by metal coordination bonds. (**D**) Cys-sGFP and His-RFP co-immobilized on AG-Co^2+^/S. From left to right: green channel (λ_ex_: 488 nm, λ_em_: filter LP505 nm), red channel (λ_ex_: 561 nm, λ_em_: filter LP565 nm), overlay of fluorescence and brightfield signals, and the scheme of the protein immobilization.

Fluorescence microscopy imaging reveals that the two fluorescent proteins were indeed co-immobilized on the same particles but showed a different spatial organization at the microscale. While His-RFP was mainly located at the outer surface of the beads, the Cys-sGFP penetrated deeper into the microstructure of beads (Figure 5). The different spatial organization of each protein relies on the immobilization rate since binding through the thiol-disulfide exchange mechanism was significantly slower than immobilization through the metal chelates. These data agree with previous results that put forth that low immobilization rates lead to more uniform distribution of proteins across the porous structure of microparticles [22]. Therefore, peptide tags serve to co-immobilize proteins in one-pot but also to tune their spatial distribution. Fine tuning of protein organization across solid materials is a technological asset to optimize biomaterials. This work demonstrates that the spatial organization can be genetically programmed by fusing different peptides tags at the N-terminus of FPs.

### 2.4. Selective Immobilization of Lys-Tagged Fluorescent Proteins on Negatively Charged Materials

**Figure 6:**
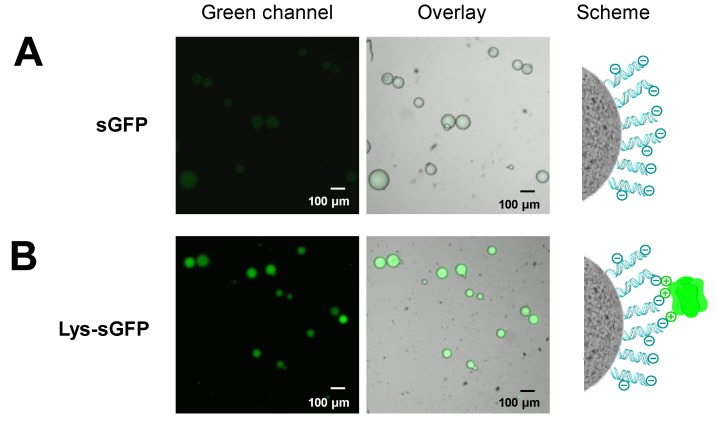
CLSM imaging of Lys-sGFP immobilized on gDNA-coated sepharose microbeads. (**A**) Untagged sGFP was incubated with agarose microbeads functionalized with DNA. (**B**) Lys-sGFP immobilized on agarose microbeads functionalized with DNA through ionic interactions. From left to right: green channel (λ_ex_: 488 nm, λ_em_: LP505 nm), overlay of fluorescence and brightfield signals, and the scheme of the protein immobilization.

Peptide tags enriched with Lys residues have been also used to control the immobilization of proteins. A Lys-tag was fused at the C-terminus of penicillin G acylase to enable its irreversible immobilization through aldehyde chemistry [32]. Furthermore, our group has recently used an *in vitro* synthesized green fluorescent protein tagged with a Lys-tag at its N-terminus (Lys-sGFP) to functionalize silica nanoparticles and glass slides [23]. Since the reversible interaction between the Lys-sGFP and the negatively-charged surfaces was demonstrated to be efficient and stable, we have applied here this principle to functionalize sepharose microbeads coated with genomic DNA (gDNA). After incubating the Lys-sGFP and the gDNA-coated microbeads for 1 h, 80% of immobilization yield was reached while only 39% of the untagged version of sGFP was immobilized on the same biofunctionalized beads (Figure 6). The unspecific immobilization of untagged sGFP may rely on the interaction between the high number of basic Lysines on the surface of sGFP (Figure S2) and the negatively-charged mesh formed by the gDNA surrounding the porous sepharose microbeads.

In this experiment, we demonstrated that the functionalization of gDNA-coated microbeads with FPs can be increased by genetically fusing a Lys-tag to the FP. This approach might enhance the light-emitting properties of DNA-coated photoactive materials which are gaining momentum in recent years. Ahn’s group found that by using double-stranded DNA as a coating agent for nanoparticles, the efficiency of the semiconductor OLEDs (organic light-emitting diode) was improved and the light intensity increased 30 times [33].

To expand the palette of photoactive materials herein presented, we exploited the Lys-sGFP variant to functionalize upconverting nanoparticles (UCNPs). This type of nanomaterial is able to harvest infrared light to emit fluorescence at lower wavelengths (in the UV-vis range). In this work, we have used core@shellNaYF_4_:Yb^3+^/Tm^3+^@NaYF_4_UCNPs previously developed by Ruggiero et al. [34] to enable the photoactivation of metal-based prodrugs under NIR light. The strategy that we adopted, exploited a good match between one of the emission bands of UCNPs (475 nm) and the major absorption wavelength of sGFP. Thus, energy transfer from the nanoparticle to the protein fluorophore is expected to result in the observation of protein fluorescence at 510 nm upon NIR light excitation.

**Figure 7:**
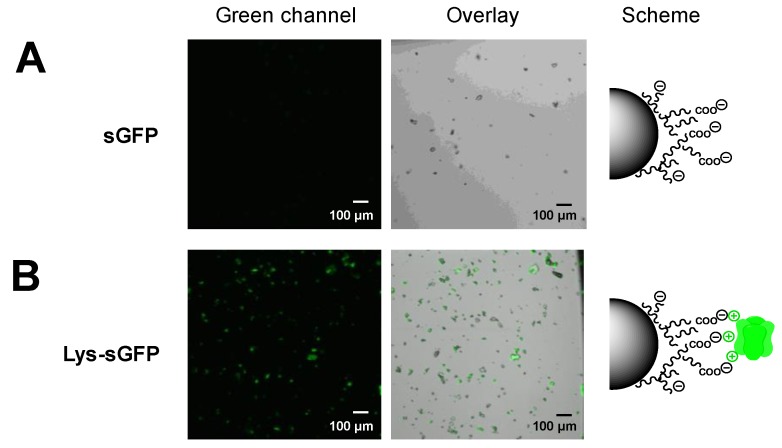
CLSM of Lys-sGFP immobilized on alginate-coated upconverting nanoparticles (UCNPs) after sample washing. (**A**) Untagged sGFP was incubated with UCNPs decorated with alginate. (**B**) Lys-sGFP immobilized on UCNPs decorated with alginate through ionic interactions. From left to right: green channel (λ_ex_: 488 nm, λ_em_: LP505 nm), overlay of fluorescence and brightfield signals, and the scheme of the protein immobilization.

To enhance such energy transfer, the protein must be localized close to the surface of the UCNPs. To this aim, we firstly immobilized both Lys-sGFP and untagged sGFP on UCNPs. Initially, we employed oleate-free core@shellNaYF_4_:Yb^3+^/Tm^3+^@NaYF_4_ UCNPs, however both fluorescent variants were barely immobilized on these nanoparticles. These results support the fact that the surface of these UCNPs is able to ionically bind negatively charged compounds such as phosphorylated molecules or anionic polymers but fails to absorb positively charged biomolecules. Therefore, to immobilize the fluorescent protein on UCNPs, we propose firstly coating their surface with an anionic polymer such as alginate and further selectively immobilizing a Lys-sGFP. The presence of carboxylic groups at the surface of UCNPs due to the alginate coating favored the ionic exchange of Lys-sGFP over the untagged protein. As result, the Lys-sGFP was immobilized on alginate-coated UCNPs with a 70% yield compared to the 30% yield found for the untagged sGFP under the same conditions (18 h in 10 mM Tris-HCl buffer at pH 7.5) (Figure S6). Furthermore, after washing the alginate-coated UCNPs, untagged sGFP was fully removed while the Lys-sGFP remained bound to the hybrid materials (Figure 7). These data demonstrate that Lys-tag mediates the immobilization on this type of hybrid materials through specific ionic interactions with the carboxylic groups grafted to the biopolymer.

**Figure 8:**
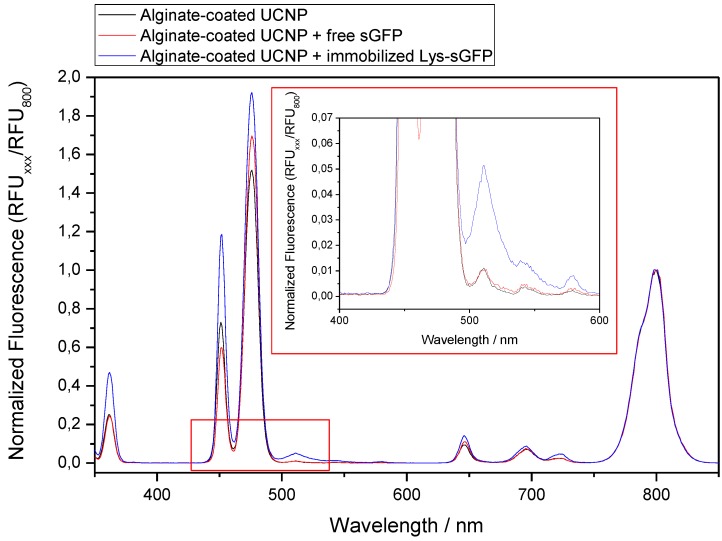
Emission spectra of alginate-coated UCNPs. All the signals were normalized assigning a value of 1 to the peak at 800 nm.

Alginate-coated UCNPs functionalized with Lys-sGFP were submitted to NIR light irradiation using a 980-nm laser and the emission spectrum was in situ recorded to measure the emitted light at 510 nm as result of the energy transfer from UCNPs to the Lys-sGFP fluorophore (Figure 8). Only when sGFP was tagged and closely immobilized on alginate-coated UCNPs, did we observe a clear emission peak at 510 nm among the characteristic emission peaks of rare-earth nanoparticles. When the coated UCNPs were incubated with the untagged protein under exactly the same conditions, the energy transfer did not occur. This experiment demonstrates that energy transfer only takes place when fluorescent protein is rather close to the surface of the UCNP, and this is only possible after functionalizing the nanoparticles with alginate and genetically fusing sGFP with a Lys-tag that selectively binds to the anionic polymer.

**Figure 9:**
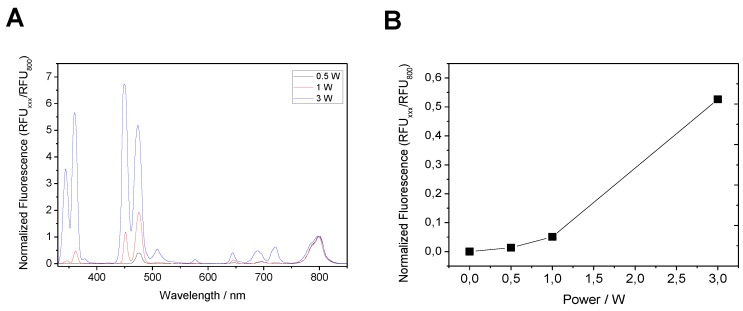
Effect of light power on the excitation of Lys-sGFP immobilized on UCNPs. (**A**) Emission spectra of immobilized Lys-sGFP on UCNPs coated with alginate at different power intensities. All the signals were normalized assigning a value of 1 to the peak at 800 nm. (**B**) Emitted fluorescence at 510 nm using different excitation intensities.

Moreover, we observed an effect of excitation light power on the efficiency of the UCNPs ability to excite the immobilized Lys-sGFP. Figure 9 indicates that at the higher power, more protein excitation is detected. Under 980 nm excitation at 3 W, the emission fluorescence intensity of Lys-sGFP immobilized on alginate-UCNPs was almost 40-fold higher than that obtained with a light power of 0.5 W. The demonstration of energy transfer between UCNPs and fluorescent proteins has rarely been reported in the literature and these preliminary results are a pioneering example of how genetically programmed proteins can be closely and selectively attached to these materials with the help of biopolymers. These biomaterials open new avenues in the photochemical application of fluorescent proteins. Excitation of fluorescent proteins with NIR light might have relevance for *in vivo* applications as well as for photonic devices activated with low energy light.

## 3. Materials and Methods

### 3.1. Materials

A six-channel μ-Slide VI ^0.4^ was purchased from ibidi (Planegg, Germany). Agarose-based functionalized materials were fabricated using plain agarose beads purchased from ABT technologies (Madrid, Spain). Epoxy-activated methacrylate beads (ECR8214F) were kindly donated by Purolite (Llantrisant, UK). The Silver stain plus^TM^ kit and Micro Bio-spin^TM^ chromatographic columns were acquired from BIORAD. TALON^®^ Metal Affinity Resin was purchased from Clontech Laboratories, Inc. 5,5′-dithiobis(2-nitrobenzoic acid), kanamycin, Triton X-100, and sodium alginate were acquired from Sigma-Aldrich (St. Louis, IL, USA).

### 3.2. Preparation of Supports

#### 3.2.1. Reduced Graphene Composite Structures Functionalized with Epoxy and Cobalt-Chelates (rGOe-Co^2+^)

The rGO self-assembly nanostructure was synthesized by reduction of GO in aqueous solution (4 mg/mL) using ascorbic acid (AsA) in a 1:1 weight ratio of GO:AsA. These nanostructures were immersed in polymer latex (polymer nanoparticles of 69 nm in aqueous suspension), produced by batch emulsion co-polymerization of methylmetactylate and glycidyl methacrylate (weight ratios of 99:1; 98:2; and 97:3). As a result, the self-assembly structures of rGO were decorated with polymer nananoparticles introducing the epoxy functionalities onto their surface (rGOe). The functionalization of rGOe with metal chelates (rGOe-Co^2+^) was performed following the procedure for the activation of epoxy-activated methacrylate resins described elsewhere [27]. Briefly, 1g of rGOe was partially modified with 500 mM IDA (iminodiacetic acid) at pH 11.0 for 24 h. Then, the flakes were washed with 100 mL of distilled water. The filtered and dried flakes were incubated with 30 mgmL^−1^ of CoCl_2_ in H_2_0 for 1 h. Finally, the material was washed with an excess of distilled water and stored at room temperature.

#### 3.2.2. Heterofunctional Cobalt- and Thiol-Activated Agarose (AG-Co^2+^/S)

The activation of this support was performed according to the procedure described in [23]. Briefly, 100 mg of epoxy agarose microbeads (6BCL) were partially modified with 500 mM IDA at pH 11.0 for 3 h. Epoxy-carboxyl was mixed with 10 mM Na_2_S (in 100 mM NaHCO_3_ at pH 10) for 1 h. The support (carboxyl-thiol) was incubated with a solution of 10 mM DTNB (in 50 mM KH_2_PO_4_ at pH 8) for 2 h. The resulting support was activated with cobalt by mixing with 30 mgmL^−1^ of CoCl_2_ in H_2_0 for at least 1 h. Finally, AG-Co^2+^/S microbeads were washed three times with 10 volumes of 10 mM Tris-HCl buffer at pH 7.5 and stored at 4 °C.

#### 3.2.3. Agarose Coated with Genomic DNA

100 mg of plain 6% cross-linked agarose microbeads (6BCL) were mixed with gDNA extracted from *E. coli* for 1 h. Then, the resulting agarose was washed three times with 10 volumes of H_2_O and stored at 4 °C.

#### 3.2.4. Upconverting Nanoparticles Coated with Alginate (UCNP-ALG)

Core@shellNaYF_4_:Yb^3+^/Tm^3+^@NaYF_4_ were synthesized and characterized as previously reported by Ruggiero et al. [34]. Oleate-free UCNPs were obtained following a reported procedure by Bogdan et al. [35]. In brief, 50 mg of nanoparticles were suspended in 5 mL of H_2_O in a round-bottom flask and pH was adjusted to 4 by using 0.1 M HCl solution. Then, the suspension was stirred for 2 h at room temperature. Afterwards, the oleate-free UCNPs were purified from the released oleic acid by extraction with diethyl ether (3 × 5 mL). The product (ca. 30 mg) was dried at room temperature overnight.

Coated UCNP with sodium alginate (UCNP-ALG) were prepared by mixing a suspension (1:10 w/v) of UCNP and sodium alginate 1% in 25 mM sodium phosphate buffer solution at pH 5 under orbital agitation for 1 h at 25 °C. Later, the UCNP-ALG were washed three times with 10 volumes of buffer and stored at 4 °C.

### 3.3. Protein Expression

The genetic constructs for the plasmid toolkit were developed in a previous work [23]. In short, a total of 1 mL of an overnight culture of *E. coli* BL21(DE3) transformed with the respective plasmid (His-sGFP_pET28b, Cys-sGFP_pET28b, Lys-sGFP_pET28b, sGFP_pET28b and His-mCherry_pET28b) was used to inoculate 50 mL of LB medium containing 30 μgmL^−1^ kanamycin. The resulting culture was incubated at 37 °C with vigorous shaking until the OD_600nm_ reached 0.6. At that point, the culture was induced with 1 mM IPTG. Cells were grown at 37 °C for 3 h and then harvested by centrifugation at 4211 *g* for 30 min at 4 °C. The resulting pellet was resuspended in 5 mL of 25 mM sodium phosphate buffer solution at pH 7. Cells were broken by sonication using a LABSONIC P, Sartorius Stedim biotech at 30% amplitude (5 s ON / 5 s OFF) for 5 min at 4 °C. The suspension was then centrifuged at 10,528 *g* for 30 min at 4 °C. Cell extracts from the supernatant containing the fluorescent protein were collected and used for future immobilizations.

### 3.4. Protein Quantification

Fluorescent proteins were quantified by measuring the fluorescence of 30 µL of cell extracts in a NUNC^TM^ 384-well black plate using a Varioskan^TM^ Flash Multimode Reader (Thermo Scientific,Waltham, MA, USA). Then, the protein content was estimated employing a calibration curve using purified sGFP and mCherry as standards.

### 3.5. Protein Immobilization

For this, 100 mg of support were incubated with 900 µL of cell extract at 0.1 mg/mL (in 25 mM sodium phosphate buffer at pH 7.5) for 1 h at room temperature with orbital shaking, unless otherwise specified. The immobilization course was followed by measuring the fluorescence in the supernatant (30 µL) using NUNC^TM^ 384-well black plates and Varioskan^TM^ Flash Multimode Reader (Thermo Scientific). Afterwards, the suspension was filtered (for graphene-based supports, TALON^®^, AG-Co^2+^/S, Purolite^®^ and gDNA-coated agarose) or centrifuged (in the case of UCNP). In all cases, the immobilization yield (Ψ) was calculated as follows:

$$\Psi \left(\%\right)=(\frac{{\mathrm{FP}}_{\mathrm{offered}}-{\mathrm{FP}}_{\mathrm{supernatant}}}{{\mathrm{FP}}_{\mathrm{offered}}})\;\times \;100$$

where the FP_initial_ is protein concentration of the fluorescent protein solution offered to the carrier, while FP_supernatant_ is the concentration of fluorescent protein that remains in the supernatant after the immobilization time.

The immobilization of sGFP on Purolite^®^ was carried out in presence of 10% Triton X-100 to avoid hydrophobic interactions. After protein immobilization, 0.1 g of beads was rinsed with 1mL of a 10% Triton X-100 solution for 5 min with orbital shaking.

### 3.6. Silver Staining of Proteins Following Polyacrylamide Gel Electrophoresis

Lys-sGFP and untagged sGFP from immobilization on UCNP-ALG were detected by highly sensitive detection using the silver staining protocol from BIORAD.

### 3.7. CLSM Microscopy Imaging

After protein immobilization, microbeads were placed on a channel of 6-channel μ-Slide VI ^0.4^. The brightfield transmission, fluorescence from sGFP (λ_ex_: 488 nm, λ_em_: filter LP505 nm) and RFP (λ_ex_: 561 nm, λ_em_: filter LP565 nm) were obtained with a confocal microscope (LSM510, ZEISS). Images were processed with FIJI (ImageJ) and ZEN 2012 (ZEISS) software.

## 4. Conclusions

We report a toolbox of plasmids and functionalized solids to fabricate a variety of genetically programmable photoactive materials. The genetic constructs encode red and green fluorescent proteins tagged to different polypeptides at their N-terminus. The aminoacid composition of such tags allows the different fluorescent variants to be selectively immobilized on different solid materials functionalized with several reactive groups such as metal chelates, disulfides, epoxy and polymeric carboxylic groups. Herein, the selective immobilization of genetically programmed fluorescent proteins was exploited for graphene-based hydrogels forming flakes, for porous microparticles based both on biopolymers such as agarose, and acrylic polymers like methacrylate, and for inorganic nanoparticles composed of rare-earth materials. Finally, we have tested the immobilized fluorescent proteins in different technological applications. On the one hand, His-tagged RFP immobilized on graphene-based material was exploited as a fluorescent pH sensor; the biomaterial worked for alkaline pHs, although its performance was significantly less efficient, and the dynamic range shorter than soluble RFP. On the other hand, Lys-tagged sGFP immobilized on alginate-coated UCNPs was employed as a photochromic material excited with IR light. The immobilization chemistries as well as the genetic construction herein developed open new technological opportunities to fabricate advanced photoactive materials with potential as light-emission devices such as bio-LEDs and biosensors.

## Figures and Tables

**Table 1:** Immobilization parameters of fluorescent proteins (FPs) immobilized on different activated supports. RFP: red fluorescent protein; sGFP: superfolder green fluorescent protein.

Functional Group	FP	Epoxy Density	Immobilization Yield (%)	Offered (mg_FP_/g_carrier_)	Immobilized (mg_FP_/g_carrier_)
None	His-RFP	High	7	2	0.14
Epoxy	sGFP	High	97	2	1.9
	His-RFP	Low	30	2	0.6
		Medium	60	2	1.2
		High	95	2	1.9
			59	22	13
			43	42	18
Epoxy and Co^2+^-chelates	sGFP	High	98	2	1.9
	His-RFP	High	99 ± 1	2	1.9
			95	22	21
			59	42	25

## Supplementary Materials

The following are available online at https://www.mdpi.com/1420-3049/24/15/2775/s1, Figure S1. SDS-PAGE of soluble fractions after cell disruption by sonication, Figure S2. X-ray of the tertiary structure of sGFP, Figure S3. SDS-PAGE of the elution of His-RFP from rGOe, Figure S4. Selective of immobilization of Cys-sGFP and His-RFP on Purolite^®^ and TALON^®^, respectively, Figure S5. Selective co-immobilization of Cys-sGFP and His-RFP on AG-Co2+/S, Figure S6. Selective of immobilization of Lys-sGFP on UCNPs.
