# Supplementary material for: Selective Immobilization of Fluorescent Proteins for the Fabrication of Photoactive Materials
Source: Molecules. 2019 Jul 30;24(15):2775. doi: 10.3390/molecules24152775 (PMC6696454; doi:10.3390/molecules24152775)
Supplement: Supplementary file 1 [file molecules-24-02775-s001.pdf]

Selective Immobilization of Fluorescent Proteins for the Fabrication of Photoactive Materials

Ana I. Benítez-Mateos, Eshan Mehravar, Susana Velasco-Lozano, Radmila Tomovska, Luca Salassa and Fernando López-Gallego

Index:

1. Expression of sGFP and RFP.
    - **Figure S1.** SDS-PAGE of soluble fractions after cell disruption by sonication.
2. Immobilization of sGFP and His-RFP on grapheme-based carriers
    - **Figure S2.** X-ray of the tertiary structure of sGFP.
    - **Figure S3.** SDS-PAGE of the elution of His-RFP from rGOe.
3. One-pot selective immobilization of Cys-sGFP and His-RFP on different materials through different immobilization chemistries
    - **Figure S4.** Selective of immobilization of Cys-sGFP and His-RFP on Purolite® and TALON®, respectively.
    - **Figure S5.** Selective co-immobilization of Cys-sGFP and His-RFP on AG-Co^{2+}/S.
4. Selective of immobilization of Lys-sGFP on UCNPs
    - **Figure S6.** Selective of immobilization of Lys-sGFP on UCNPs

1. Expression of sGFP and RFP

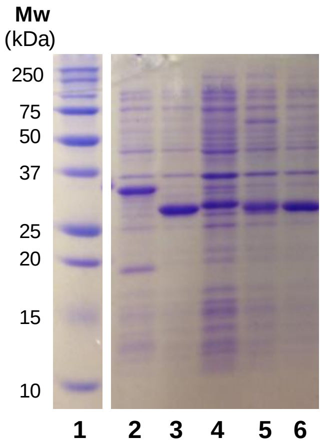

Figure S1. SDS-PAGE of soluble fractions after cell disruption by sonication. (1) Molecular weight markers (BioRad Precision Plus Protein All Blue Standard); (2) His-RFP, (3) untagged sGFP, (4) Lys-sGFP, (5) Cys- sGFP, (6) His- sGFP.

2. Immobilization of sGFP and His-RFP on grapheme-based carriers

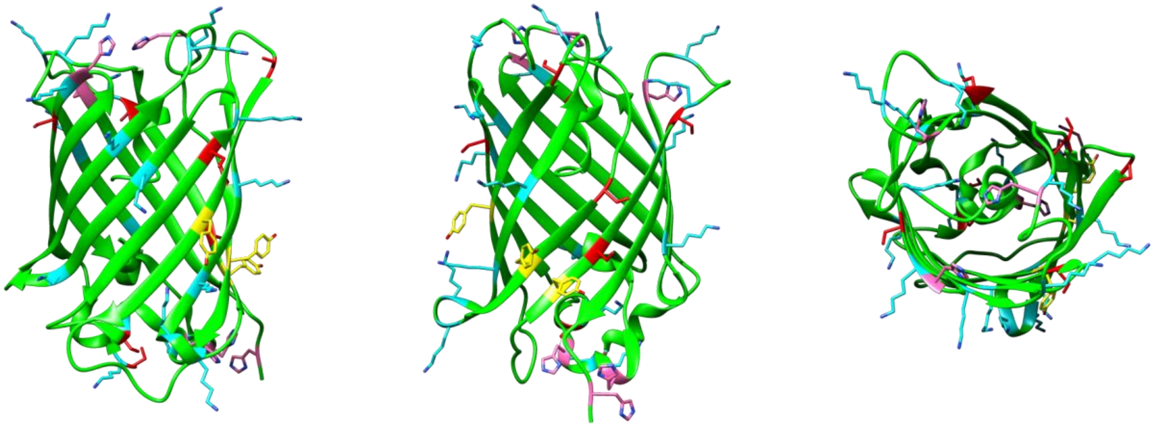

Figure S2. X-ray of the tertiary structure of sGFP. Three different views are represented. The main reactive and exposed residues are highlighted: 19 Lysines (cyan), 8 Serines (red), 5 Histidines (pink) and 3 Tyrosines (yellow).

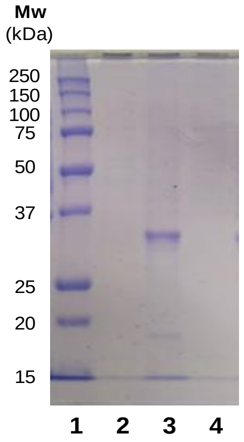

Figure S3. SDS-PAGE of the elution of His-RFP from rGOe. (1) Molecular weight markers (BioRad Precision Plus Protein All Blue Standard); (2) rGOe with high epoxy density, (3) rGOe with low epoxy density, (4) rGOe with medium epoxy density.

3. One-pot selective immobilization of Cys-sGFP and His-RFP on different materials through different immobilization chemistries

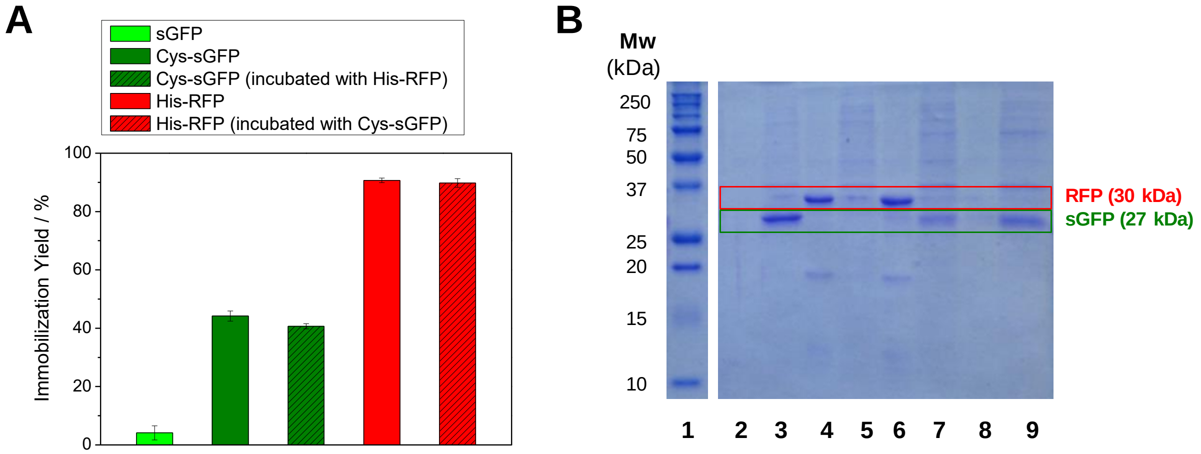

Figure S4. Selective immobilization of Cys-sGFP and His-RFP on Purolite® and TALON®, respectively. (**A**) Immobilization yields of untagged sGFP (green bar), Cys-sGFP (dark green bar) and Cys-sGFP incubated with His-RFP (dashed dark green bar) and Purolite®; His-RFP (red bar) and His-RFP incubated with Cys-sGFP (dashed red bar) and TALON®. The immobilization yields were calculated after 1 hour of incubation by measuring the fluorescence in the supernatant in a plate-reader. (**B**) SDS-PAGE of the different immobilization reactions. (1) Molecular weight markers (BioRad Precision Plus Protein All Blue Standard); (2) untagged sGFP immobilized on Purolite® and TALON® ; (3) untagged sGFP from the supernatant after incubation with Purolite® and TALON®; (4) His-RFP immobilized on TALON®; (5) His-RFP from the supernatant after incubation with Purolite® and TALON®; (6) Cys-sGFP immobilized on Purolite® and His-RFP immobilized on TALON®; (7) Cys-sGFP and His-RFP from the supernatant after incubation with Purolite® and TALON®; (8) Cys-sGFP immobilized on Purolite®; (9) Cys-sGFP from the supernatant after incubation with Purolite® and TALON®. The samples of lines (2), (4), (6) and (8) correspond to

the microbeads with the proteins immobilized. The samples of lines (3), (5), (7) and (9) are the supernatant after the protein immobilization and the filtration step.

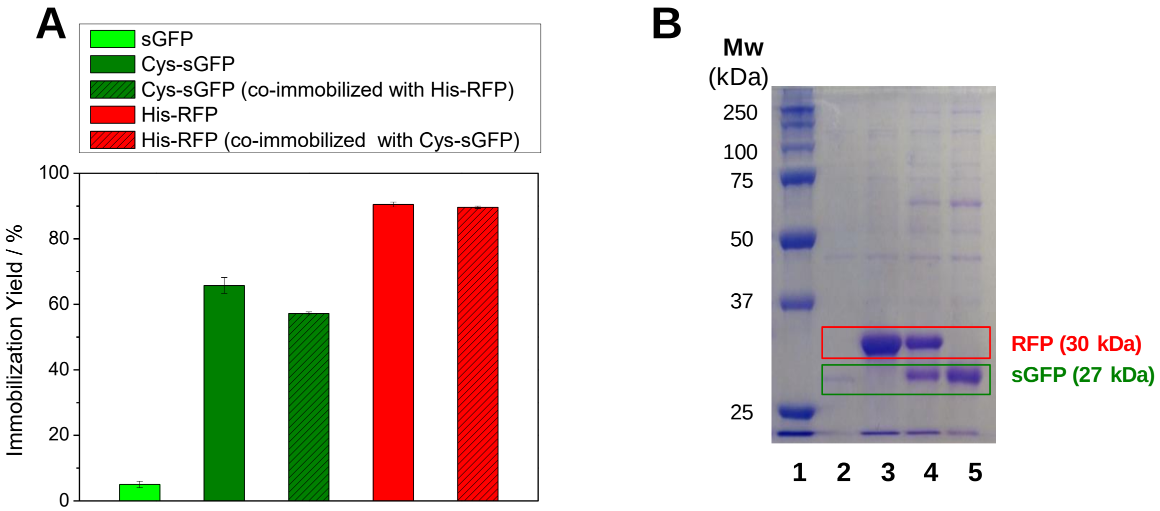

Figure S5. Selective co-immobilization of Cys-sGFP and His-RFP on AG-Co^{2+}/S. (**A**) Immobilization yields after incubation of untagged sGFP (green bar), Cys-sGFP (dark green bar), Cys-sGFP co-immobilized with His-RFP (dashed dark green bar), His-RFP (red bar) and His-RFP incubated with Cys-sGFP (dashed red bar)on AG-Co^{2+}/S. The immobilization yields were calculated after 1 hour of incubation by measuring the fluorescence in the supernatant in a plate-reader. (**B**) SDS-PAGE of the different immobilization reactions. (1) Molecular weight markers (BioRad Precision Plus Protein All Blue Standard); (2) untagged sGFP immobilized on AG-Co^{2+}/S; (3) His-RFP immobilized on AG-Co^{2+}/S; (4) His-RFP and Cys-sGFPco-immobilized on AG-Co^{2+}/S; (5) Cys-sGFP immobilized on AG-Co^{2+}/S. The samples of lines (2), (3), (4) and (5) correspond to the microbeads with the proteins immobilized after the filtration step.

4. Selective of immobilization of Lys-sGFP on UCNPs

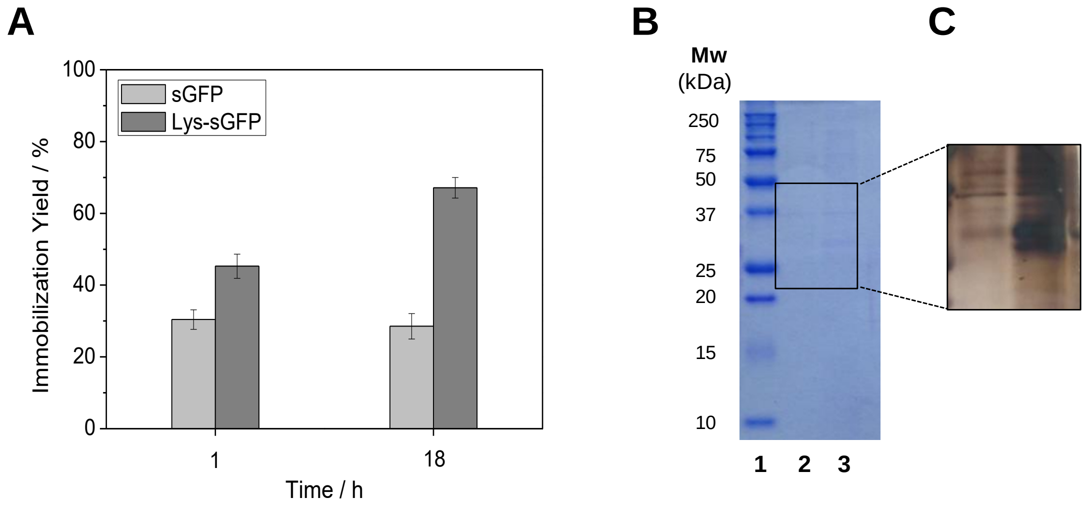

Figure S6. Selective immobilization of Lys-sGFP on UCNPs. (**A**) Immobilization yields of sGFP and Lys-sGFP immobilized on UCNPs. The immobilization yields were calculated after 1 hour of incubation by measuring the fluorescence in the supernatant in a plate-reader. (**B**) SDS-PAGE of the immobilized proteins on UCNP-ALG. (1) Molecular weight markers (BioRad Precision Plus Protein All Blue Standard); (2) sGFP; (3) Lys-sGFP. (**C**) Silver staining of the SDS-PAGE.
